# Evaluation of the blood-oxygen-level-dependent (BOLD) sequence with 3 Tesla device in renal transplant patients in the assessment of early allograft disfunction, correlated with biopsy

**DOI:** 10.31744/einstein_journal/2021AO6069

**Published:** 2021-08-12

**Authors:** Guilherme Falleiros Mendes, Priscila Mina Falsarella, Rodrigo Gobbo Garcia, Liana Guerra Sanches, Ronaldo Hueb Baroni

**Affiliations:** 1 Hospital Israelita Albert Einstein São PauloSP Brazil Hospital Israelita Albert Einstein, São Paulo, SP, Brazil.

**Keywords:** Magnetic resonance imaging, Kidney transplantation, Biopsy, Primary graft dysfunction

## Abstract

**Objective:**

To evaluate the ability of blood-oxygen-level-dependent (BOLD) magnetic resonance imaging at 3 Tesla to measure tissue oxygen bioavailability based on R2* values, and to differentiate between acute tubular necrosis and acute rejection compared to renal biopsy (gold standard).

**Methods:**

A prospective, single-center study, with patients submitted to renal transplantation between 2013 and 2014, who developed graft dysfunction less than 4 weeks after transplantation. All patients were submitted to abdominal magnetic resonance imaging at 3 Tesla using the same protocol, followed by two BOLD sequences and kidney biopsy.

**Results:**

Twelve male (68.75%) and three female (31.25%) patients were included. A total of 19 percutaneous renal biopsies were performed (four patients required a second biopsy due to changes in clinical findings). Pathological findings revealed ten cases of acute tubular necrosis, four cases of acute rejection, and five cases with other (miscellaneous) diagnoses. Comparison between the four groups of interest failed to reveal significant differences (p=0.177) in cortical R2* values, whereas medullary R2* values differed significantly (p=0.033), with lower values in the miscellaneous diagnoses and the acute tubular necrosis group.

**Conclusion:**

BOLD magnetic resonance imaging at 3 Tesla is a feasible technique that uses indirect tissue oxygen level measurements to differentiate between acute rejection and acute tubular necrosis in renal grafts.

## INTRODUCTION

Kidney transplantation is the ideal renal replacement therapy for chronic renal failure, with surgical outcomes and graft durability having improved significantly in recent years.^( 1 )^ Allograft dysfunction after transplantation is relatively common and may lead to graft loss in the long-term. Acute tubular necrosis (ATN) and acute rejection (AR) are common causes of early allograft dysfunction, which may be difficult to differentiate.^( 2 )^ Conventional diagnostic protocols based on clinical data, laboratory tests and Doppler ultrasound (US) are not specific for differentiating between ATN and AR. Therefore, US guided biopsy is the gold standard for differential diagnoses of these conditions.^( 2 )^

Although rare, complications associated with kidney biopsy are potentially severe, the most frequent being bleeding, which requires transfusion in up to 1% of cases, arteriovenous fistulas, and eventual allograft loss.^( 3 )^

The effectiveness of blood-oxygen-level-dependent (BOLD) magnetic resonance imaging (MRI) in assessing pathological conditions, such as renal artery stenosis, ureteral obstruction, diabetic nephropathy, AR and ATN, has been demonstrated in many recent studies.^( 4 )^

BOLD MRI is a noninvasive modality that evaluates tissue oxygen concentration. In this technique, the paramagnetic effect of deoxyhemoglobin acts as an endogenous contrast agent.^( 5 )^ When tissue oxygen concentration drops, tissue deoxyhemoglobin concentration increases, leading to signal reduction in the T2* sequence and resultant increase in the apparent relaxation rate R2* (1/T2*).^( 5 )^

In normal kidneys, oxygen concentration is slightly higher in the cortex than in the medulla, and this difference that can be detected using BOLD MRI.^( 5 )^ Patients with acute kidney allograft rejection show a significant increase in oxygen concentration in the medulla, seen as a decrease in the R2* signal.^( 5 , 6 )^

Most studies using BOLD MRI at 1.5 Tesla to differentiate between AR and ATN have shown good results. However, few studies have used 3 Tesla (3T) magnets, which allow for higher spatial resolution and better visual differentiation between the renal cortex and medulla.^( 1 , 5 , 7 , 8 )^

## OBJECTIVE

To evaluate the ability of BOLD magnetic resonance imaging at 3 Tesla to measure tissue oxygen bioavailability based on R2* values, and to differentiate between acute tubular necrosis and acute rejection compared to renal biopsy (gold standard).

## METHODS

### Patients

This prospective single-center study was conducted with consecutive male and female patients aged over 18 years, who underwent renal transplantation between 2013 to 2014, and developed graft dysfunction less than four weeks after transplantation. Exclusion criteria were graft dysfunction due to vascular anastomosis, ureteral anastomosis, or extrinsic compression by fluid collections, and contraindications to MRI.

Patients were allocated to one of two groups. Group 1 comprised patients with laboratory diagnosis of allograft dysfunction (use of serum urea and creatinine levels to estimate renal function with the Cockcroft-Gault formula). Group 2 (Control Group) comprised patients with similar demographic characteristics, who also underwent renal transplantation between 2013 to 2014, but did not develop allograft dysfunction less than four weeks after transplantation according to the same evaluation criteria.

Groups 1 and 2 patients were submitted to abdominal MRI at 3T using the same protocol. Within the next 12 hours, Group 1 patients were also submitted to US-guided allograft biopsy to obtain a definitive diagnosis of renal dysfunction and guide appropriate treatment. Group 1 patients were subsequently subdivided into three subgroups according to anatomopathological findings and compared with the Group 2.

This study was approved by the Research Ethics Committee of *Hospital Israelita Albert Einstein* (HIAE), protocol 502.141, CAAE: 13521313.0.0000.0071. All patients signed an informed consent form.

### Magnetic resonance imaging

Magnetic resonance imaging was performed using a 3T magnet (Magnetom Prisma Trio, Siemens Healthineers, Erlangen, Germany). Magnetic resonance imaging assessments included T1- and T2-weighted imaging for anatomical distinction, followed by diffusion-weighted imaging and two BOLD sequences. In BOLD MRI, images were acquired in the coronal plane using three-dimensional multi-echo gradient-echo sequences, with 6 and 8 echoes. Echo time (ET) and repetition time (RT) corresponded to 1.2 to 7.5ms and 0.9 to 9.7ms and 9ms and 11.3ms (6 and 8 gradient-echo sequence respectively). Other settings were slice thickness of 4mm, field of view of 34 to 37cm and 256x256 matrix.

Cortical and medullary R2* (=1/T2*) rates were measured in BOLD images at three different sites (upper, mid and lower poles of the renal cortex and medulla) in regions of interest measuring 1cm. Medians were used in the analysis,^( 5 )^ and the cortical/medullary ratio was also calculated. R2* measurements were qualitatively analyzed using color maps. High deoxyhemoglobin levels (high signal intensity in R2*) were shown in red. Low deoxyhemoglobin levels (low signal intensity in R2*) were shown in yellow. Associations between R2* rate and biopsy classification (ATN or AR) were investigated.

Magnetic resonance images were independently interpreted by two experienced (>10 years) abdominal radiology specialists blinded to patient clinical and laboratory data, and the diagnosis obtained by consensus agreement.

### Biopsy and histopathological findings

Biopsy specimens were collected by one of 8 experienced (>5 years) interventional radiologists using aseptic techniques and local anesthesia (2% lidocaine hydrochloride). Procedures were ultrasound-guided (iU22 Matrix US scanner, Philips Healthcare, Andover, MA, USA; Aplio™ 500 Platinum Series, Toshiba American Medical Systems, Tustin, CA, USA or LOGIQ E9 VNav GE Healthcare, Milwaukee, WI, USA) and two to three fragments collected. Patients were observed and their vital signs monitored during anesthetic recovery. Patients were submitted to sonographic assessment of the puncture site within the first hour after biopsy collection and remained in bed rest for 4 to 6 hours.

Biopsy fragments were divided into two samples. One sample was fixed in Bouin’s solution for optical microscopy, and the other in Michel’s solution for immunofluorescence. Slides were prepared, stained with periodic acid-Schiff (PAS), Masson’s trichrome, picrosirius, and Jones methenamine silver, and analyzed by one of three urologic pathology specialists.

### Statistical analysis

Categorical variables were described as absolute frequencies and percentages. The distribution of numerical variables was examined using boxplots and histograms and tested for normality using the Shapiro Wilk test. Given their non-normal distribution, numerical variables were described as median, interquartile range, and minimum and maximum values. Intergroup comparisons were performed using the χ^2^ test or the Fisher’s exact test for categorical variables, or the non-parametric Kruskall-Wallis test for numerical variables.

Statistical analyses were performed using (SPSS) software, version 20.0. The level of significance was set at 5%.

## RESULTS

Group 1 comprised 12 male (68.75%) and three female (31.25%) patients, whereas Group 2 had four male (66.7%) and two female (33.3%) patients. The median age of Group 1 and Group 2 patients was 47.7±12.5 years (28 to 68 years) and 31.5±5.8 years (27 to 40 years), respectively. All patients in this sample (Groups 1 and 2) underwent deceased donor kidney transplantation.

**Figure 1:**
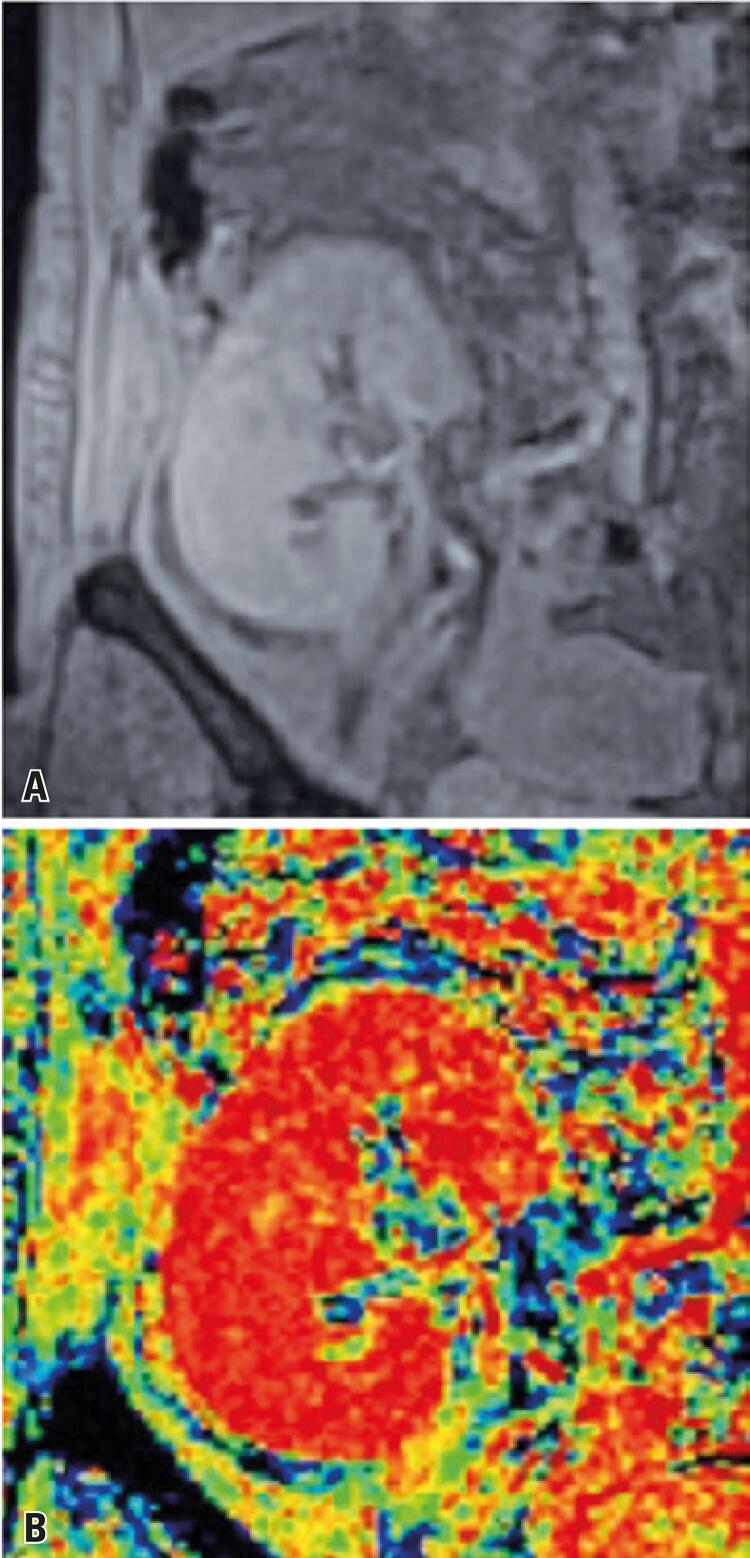
Patient with acute tubular necrosis. A) Reduction of corticomedullary differentiation; B) Note poor differentiation between cortex and medulla (color scale)

**Figure 2:**
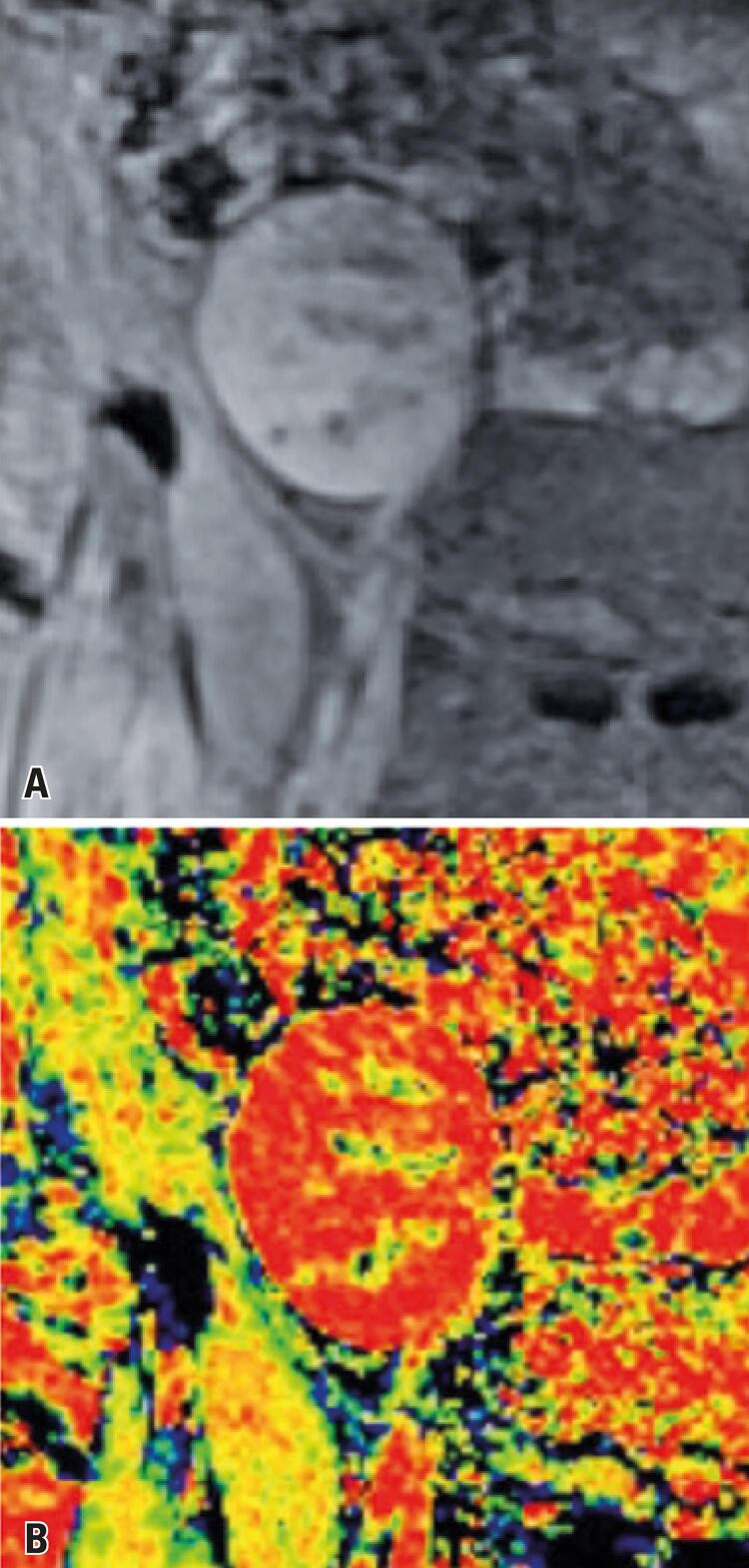
Patient with acute rejection. A) Increased corticomedullary differentiation; B) Note lower deoxyhemoglobin levels in the medulla and enhanced differentiation between the cortex and the medulla (color scale)

A total of 19 percutaneous renal biopsies were performed in Group 1 patients. Four patients required a second biopsy at a different time, due to changes in clinical findings. In such cases, a second BOLD MRI assessment was carried out prior to biopsy collection. The median time between kidney transplant and biopsy collection was 12 (4 to 25) days. Histologic analysis revealed ten cases of ATN, four cases of AR. Other five cases had different diagnoses (three cases of glomerular sclerosis, one case of cortical infarction and one case of avascular infarction), and were excluded from the analysis. Radiological findings of ATN and AR are shown in figures 1 and 2 , respectively.

Median cortical and medullary R2* rates of patients are shown in table 1 .

**Tabela 1:** Mediana do índice R2* do córtex e da medula em diferentes segmentos renais

R2*	Median (1Q-3Q)	Minimum-maximum
Upper cortex	149 (144-168)	107-182
Mid cortex	144 (134-160)	118-197
Lower cortex	155 (143-169)	102-191
Córtex	149 (139-162)	107-197
Upper medulla	201 (167-232)	116-712
Mid medulla	169 (155-210)	120-789
Lower medulla	178 (169-227)	127-468
Medulla	177 (163-219)	116-789

Q: quartile.

Patients were divided into three subgroups of interest (Control, AR and ATN), according to BOLD MRI findings. Cortical R2* rates did not differ significant (p=0.225) between subgroups. Medullary R2* rates were significantly (p=0.047) lower in the ATN relative to remaining subgroups ( Table 2 ).

**Tabela 2:** Comparação entre os grupos de acordo com os valores R2* obtidos no córtex e na medula

R2*	Group median (1Q-3Q)			p value

	Control	ATN	AR	
Cortex	155 (151-158)	143 (134-162)	164 (154-173)	0,225
Medulla	202 (186-219)	164 (154-185)	237 (206-452)	0,047

χ^2^, Fisher’s exact, and Kruskal-Wallis tests.

Q: quartile; ATN: acute tubular necrosis; AR: acute rejection.

## DISCUSSION

This study examined the applicability of BOLD MRI at 3T to assess kidney function following kidney transplantation, based on comparative analysis of patients with and without kidney changes. Imaging assessment of renal graft cortical and medullary oxygen status revealed significant (p=0.047) differences in medullary R2* rates between the AR, the ATN and the Control Groups. These differences can be explained by increased cortical and medullary deoxyhemoglobin levels in patients with ATN, which leads to a decrease in the signal of R2* and resultant loss of differentiation between these regions. In contrast, patients with AR have lower medullary deoxyhemoglobin levels, which leads to an increase in the signal of R2*, with good differentiation between the cortex and the medulla.

Echo times in this study were shorter than those reported in literature.^( 5 , 9 , 10 )^The sequence was thought to be sensitive enough to provide adequate contrast resolution in spite of shorter ET, with the benefit of shorter sequence RT and, therefore, duration.

In a study comparing patients with normal renal graft and patients with AR or ATN, Han et al.,^( 2 )^ showed differences in oxygen bioavailability in transplanted kidneys during the initial stages of allograft dysfunction can be effectively detected, using R2* rates measured in BOLD MRI.

Djamali et al.,^( 9 )^ described lower R2* rates in ATN and AR compared to normal functioning allografts, which reflects significant increases in medullary oxygen bioavailability in ATN allografts. Different from that study, this analysis revealed similar R2* rates in the ATN and the Control Group, although values were lower relative to the AR Group.

This study has some limitations. First, sample size was relatively small. Second, the fact that four patients required a second biopsy at a different time, within the first four weeks post-transplantation, due to changes in clinical findings. They were submitted to another BOLD MRI prior to the second biopsy, which may have introduced a bias in the analysis. Finally, a minority of patients (four cases) developed AR, and interobserver variability in R2* rate estimation was not assessed.

## CONCLUSION

Findings of this study suggest blood-oxygen-level-dependent magnetic resonance imaging performed on 3 Tesla magnets is a feasible technique that uses indirect tissue oxygen level measurements to detect renal injury in renal grafts. Future routine use of this type of sequence in cases of renal graft dysfunction may decrease the number of biopsies in these already debilitated patients and avoid potential complications.
