# Supplementary material for: Evaluation of the blood-oxygen-level-dependent (BOLD) sequence with 3 Tesla device in renal transplant patients in the assessment of early allograft disfunction, correlated with biopsy
Source: Einstein (Sao Paulo). 2021 Aug 12;19:eAO6069. doi: 10.31744/einstein_journal/2021AO6069 (PMC8362909; doi:10.31744/einstein_journal/2021AO6069)
Supplement: Translation

# Translation: Avaliação da sequência dependente do nível de oxigênio no sangue (BOLD) com dispositivo 3 Tesla em pacientes transplantados renais na avaliação da disfunção de enxerto precoce, correlacionada com biópsia

**Authors:** Guilherme Falleiros Mendes, Priscila Mina Falsarella, Rodrigo Gobbo Garcia, Liana Guerra Sanches, Ronaldo Hueb Baroni

**Affiliations:** 1 Brasil Hospital Israelita Albert Einstein, São Paulo, SP, Brasil.

**Keywords:** Imagem por ressonância magnética, Transplante de rim, Biópsia, Disfunção primária do enxerto

## Abstract

**Objetivo:**

Avaliar a sequência de ressonância magnética *blood-oxygen-level-dependent* (BOLD) realizada em um equipamento 3 Tesla para medir a biodisponibilidade do oxigênio do tecido pelo valor de R2* na diferenciação de necrose tubular aguda e rejeição aguda, comparando à biópsia renal (padrão-ouro).

**Métodos:**

Estudo unicêntrico, prospectivo, com pacientes submetidos a transplante renal de 2013 a 2014, que desenvolveram disfunção do enxerto menos de 4 semanas após o transplante. Todos os pacientes foram submetidos à ressonância magnética abdominal 3 Tesla com o mesmo protocolo, seguida de duas sequências BOLD e biópsia renal.

**Resultados:**

Foram incluídos 12 homens (68,75%) e três mulheres (31,25%). Foram realizadas 19 biópsias renais percutâneas (quatro pacientes necessitaram de segunda biópsia devido à alteração nos achados clínicos). Os resultados histopatológicos incluíram dez casos de necrose tubular aguda, quatro de rejeição aguda e cinco casos de outros diagnósticos (miscelânea). A comparação entre os quatro grupos de interesse não mostrou diferenças significativas em relação ao R2* no córtex (p=0,177). Quanto ao R2* da medula, observaram-se diferenças significativas (p=0,033), com miscelânea e necrose tubular aguda apresentando valores mais baixos quando comparados aos demais.

**Conclusão:**

A ressonância magnética BOLD 3 Tesla é uma técnica viável que indiretamente utiliza a concentração de oxigênio no tecido em enxertos renais e pode ser usada para a diferenciação entre rejeição aguda e necrose tubular aguda.

## INTRODUÇÃO

O transplante renal é a forma ideal de terapia de substituição renal para insuficiência renal crônica, e tanto os resultados cirúrgicos como a durabilidade do enxerto melhoraram muito nos últimos anos.^( 1 )^A disfunção do aloenxerto após o transplante é relativamente comum e pode levar à perda do enxerto no longo prazo. A necrose tubular aguda (NTA) e a rejeição aguda (RA) são causas comuns de disfunção precoce do aloenxerto, podendo ser de difícil diferenciação.^( 2 )^Os protocolos diagnósticos convencionais, baseados em dados clínicos, exames laboratoriais e ultrassonografia (US) com Doppler, não são específicos para a diferenciação entre NTA e RA, o que faz da biópsia guiada por US o exame padrão-ouro no diagnóstico diferencial dessas patologias.^( 2 )^

Embora raras, as complicações associadas à biópsia renal são potencialmente graves, sendo as mais comuns a hemorragia (com necessidade de transfusão em até 1% dos casos), as fístulas arteriovenosas e possível perda do aloenxerto.^( 3 )^

A efetividade da sequência *blood-oxygen-level-dependent* (BOLD) de ressonância magnética (RM) na avaliação de diversas doenças, como estenose da artéria renal, obstrução ureteral, nefropatia diabética, NTA e RA, foi demonstrada em diversos estudos.^( 4 )^

A sequência BOLD é um método não invasivo, que avalia a concentração tecidual de oxigênio por meio do efeito paramagnético da desoxihemoglobina, que atua como agente endógeno de contraste.^( 5 )^A queda da concentração tecidual de oxigênio aumenta a concentração tecidual de desoxihemoglobina, que reduz o sinal na sequência T2* e, por sua vez, eleva o valor aparente de relaxamento R2* (1/T2*).^( 5 )^

No rim normal, a concentração de oxigênio é um pouco mais alta no córtex do que na medula, e essa diferença pode ser detectada na sequência BOLD.^( 5 )^Em pacientes com RA do aloenxerto, ocorre aumento significativo da concentração de oxigênio na medula, levando à redução do sinal de R2*.^( 5 , 6 )^

A maioria dos estudos que empregaram a sequência BOLD em equipamentos de 1.5 Tesla para diferenciação entre RA e NTA mostrou bom resultados. Entretanto, poucos estudos utilizaram equipamentos de 3 Tesla (3T), que proporcionam resolução espacial mais alta e melhor diferenciação visual entre o córtex e a medula.^( 1 , 5 , 7 , 8 )^

## OBJETIVO

Avaliar a capacidade da sequência de ressonância magnética BOLD executada em um equipamento de ressonância 3 Tesla de mensurar da biodisponibilidade de oxigênio tecidual com base no valor R2* e de diferenciar entre a necrose tubular aguda e a rejeição aguda, comparativamente à biópsia renal (padrão-outro).

## MÉTODOS

### Pacientes

Este estudo prospectivo unicêntrico foi realizado com pacientes consecutivos de ambos os sexos e idade superior a 18 anos, submetidos a transplante renal entre 2013 e 2014 e que desenvolveram disfunção do enxerto menos de 4 semanas após o transplante. Os critérios de exclusão foram disfunção do enxerto causada por anastomose vascular, anastomose ureteral ou compressões extrínsecas por coleções, além de contraindicações para RM.

Os pacientes foram divididos em dois grupos. O Grupo 1 incluiu pacientes com diagnóstico laboratorial de perda de função do aloenxerto (uso de dosagens séricas de ureia e creatinina para estimar a função renal por meio da fórmula de Cockcroft-Gault). O Grupo 2 (Grupo Controle) incluiu pacientes com características demográficas semelhantes às do Grupo 1, também transplantados entre 2013 e 2014, mas que não desenvolveram disfunção do aloenxerto menos de 4 semanas após o transplante, segundo os mesmos critérios de avaliação.

Todos os pacientes (Grupos 1 e 2) foram submetidos à RM abdominal em equipamento de 3T e empregando-se o mesmo protocolo. Após o estudo de imagem, os pacientes do Grupo 1 foram submetidos à biópsia do aloenxerto guiada por US, realizada dentro de um intervalo máximo de 12 horas, para fins de diagnóstico definitivo de disfunção renal e direcionamento do tratamento adequado. O Grupo 1 foi, então, subdividido em três subgrupos, de acordo com os achados histológicos da biópsia, e comparado com o Grupo 2.

Este estudo foi aprovado pelo Comitê de Ética em Pesquisa do Hospital Israelita Albert Einstein (HIAE), protocolo número 502.141, CAAE: 13521313.0.0000.0071. Todos os pacientes assinaram um Termo de Consentimento Livre e Esclarecido.

### Ressonância magnética

Os exames de RM foram realizados em um equipamento de 3T (Magnetom Prisma Trio, Siemens Healthineers, Erlangen, Alemanha). Em todos os casos, imagens ponderadas em T1 e T2 foram adquiridas para diferenciação anatômica, além de uma imagem ponderada por difusão e duas sequências BOLD. Ambas as imagens BOLD foram adquiridas no plano coronal, empregando-se sequências tridimensionais multieco gradiente-eco, com 6 e 8 ecos. A sequência gradiente eco com 6 ecos foi obtida com tempo de eco (TE) de 1,2 a 7,5ms e tempo de repetição (TR) de 9ms. Na sequência com 8 ecos, foram utilizados TE de 0,9 a 9,7ms e TR de 11,3ms. Outros parâmetros avaliados foram espessura de corte de 4mm, campo de visão de 34 a 37cm e matriz de 256x256.

Os índices R2* (=1/T2*) do córtex e da medula foram mensurados na sequência BOLD em três locais diferentes (polo superior, médio e inferior do córtex e da medula), em região de interesse de 1cm. Foram calculadas as medianas das mensurações^( 5 )^e o índice cortical/medular. As mensurações realizadas em R2* foram analisadas de forma qualitativa empregando-se mapas de cores, em que as concentrações altas (alto sinal na R2*) e baixas (baixo sinal na R2*) de desoxihemoglobina foram representadas pelas cores vermelho e amarelo, respectivamente. As associações entre o índice R2* e a classificação baseada em biópsia (NTA ou RA) foram investigadas.

As imagens de RM foram interpretadas de forma independente por dois radiologistas experientes (>10 anos de experiência) especializados em radiologia abdominal, e o diagnóstico foi baseado em consenso. Os radiologistas não tiveram acesso aos dados clínicos e laboratoriais dos pacientes.

### Biópsia e achados histopatológicos

As biópsias foram coletadas por um dos oito especialistas em radiologia intervencionista experientes (>5 anos de experiência), empregando-se técnica asséptica e anestesia local (cloridrato de lidocaína a 2%). Os procedimentos foram guiados por US (iU22 Matrix US scanner, Philips Healthcare, Andover, MA, Estados Unidos; Aplio™ 500 Platinum Series, Toshiba American Medical Systems, Tustin, CA, Estados Unidos ou LOGIQ E9 VNav, GE Healthcare, Milwaukee, WI, Estados Unidos), coletando-se dois a três fragmentos por biópsia. Após os procedimentos, os pacientes permaneceram sob observação e tiveram seus sinais vitais monitorados durante a recuperação anestésica. Na primeira hora após a coleta da biópsia, os pacientes foram submetidos à avaliação por US do local de punção, permanecendo em repouso no leito por 4 a 6 horas.

Os fragmentos de biópsia foram divididos em duas amostras. Uma das amostras foi fixada em solução de Bouin para microscopia óptica e a outra, em solução de Michel, para análise por imunofluorescência. As lâminas foram preparadas, coradas com ácido periódico de Schiff (PAS - *periodic acid-Schiff* ), tricrômico de Masson, picrossírio e prata de Jones, sendo analisadas por um dos três patologistas especialistas em urologia.

### Análise estatística

As variáveis categóricas foram descritas como frequências absolutas e percentagens. A distribuição das variáveis numéricas foi avaliada por meio de gráficos de caixa e histogramas, sendo testada para normalidade por meio do teste de Shapiro-Wilk. Uma vez que as variáveis numéricas não seguiram uma distribuição normal, elas foram descritas na forma de mediana e intervalo interquartil, além de valores mínimos e máximos. As comparações entre os grupos foram realizadas por meio do teste do χ^2^e do teste exato de Fisher, para variáveis categóricas, ou do teste não paramétrico de Kruskall-Wallis, para variáveis numéricas.

As análises foram realizadas empregando-se o programa (SPSS), versão 20.0. O nível de significância adotado foi de 5%.

## RESULTADOS

O Grupo 1 incluiu 12 pacientes do sexo masculino (68,75%) e três do sexo feminino (31,25%), e o Grupo 2 contou com quatro pacientes do sexo masculino (66,7%) e dois do sexo feminino (33,3%). A mediana de idade dos pacientes do Grupo 1 foi 47,7±12,5 anos (28 a 68 anos) e a dos pacientes do Grupo 2 foi 31,5±5,8 anos (27 a 40 anos). Todos os pacientes (Grupos 1 e 2) foram submetidos a transplante renal de doador falecido.

Dezenove biópsias percutâneas foram realizadas nos pacientes do Grupo 1. Em quatro pacientes, foi necessário coletar uma segunda biópsia em outro momento, devido a alterações nos achados clínicos. Nesses casos, a biópsia foi precedida por uma nova sequência BOLD de RM. A mediana de tempo entre o transplante e a biópsia foi de 12 dias (4 a 25 dias). Os resultados histopatológicos desses pacientes revelaram dez casos de NTA e quatro de RA. Outros cinco casos receberam diagnósticos distintos (três casos de esclerose glomerular, um caso de infarto da cortical e um caso de infarto avascular) e foram excluídos da análise. Os achados radiológicos dos casos de NTA e RA podem ser visualizados nas figuras 1 e 2 , respectivamente.

**Figura 1:** Paciente com necrose tubular aguda. A) Redução da diferenciação corticomedular; B) Córtex e medula mal diferenciados (escala de cores)

**Figura 2:** Paciente com rejeição aguda. A) Aumento da diferenciação corticomedular; B) Observa-se concentração mais baixa de desoxihemoglobina na medula, com melhor diferenciação entre córtex e medula (escala de cores)

As medianas de valores R2* do córtex e da medula dos pacientes avaliados encontram-se listadas na tabela 1 .

**Tabela 1:** Mediana do índice R2* do córtex e da medula em diferentes segmentos renais

R2*	Mediana (1Q-3Q)	Mínimo-máximo
Córtex superior	149 (144-168)	107-182
Córtex médio	144 (134-160)	118-197
Córtex inferior	155 (143-169)	102-191
Córtex	149 (139-162)	107-197
Medula superior	201 (167-232)	116-712
Medula média	169 (155-210)	120-789
Medula inferior	178 (169-227)	127-468
Medula	177 (163-219)	116-789

Q: quartil.

Os pacientes foram subdivididos em três grupos de interesse (Controle, RA e NTA), de acordo com os achados da sequência BOLD de RM. Os valores de R2* do córtex não diferiram de forma significativa (p=0,225) entre os subgrupos. Os valores de R2* da medula foram significativamente (p=0,047) menores no grupo NTA em relação aos demais subgrupos ( Tabela 2 ).

**Tabela 2:** Comparação entre os grupos de acordo com os valores R2* obtidos no córtex e na medula

R2*	Grupo mediana (1Q-3Q)			Valor de p

	Controle	NTA	RA	
Córtex	155 (151-158)	143 (134-162)	164 (154-173)	0,225
Medula	202 (186-219)	164 (154-185)	237 (206-452)	0,047

Teste do χ^2^, teste exato de Fisher e teste de Kruskal-Wallis.

Q: quartil; NTA: necrose tubular aguda; RA: rejeição aguda.

## DISCUSSÃO

Este estudo avaliou a utilidade de sequências BOLD de RM obtidas com equipamentos de 3T para avaliação da função renal após o transplante renal, por meio da comparação de pacientes com e sem alterações renais. A avaliação da oxigenação da cortical e da medular do enxerto renal mostrou diferenças significativas (p=0,047) de valores R2* entre os grupos RA, NTA e Controle. Essas diferenças podem ser explicadas pelo aumento da concentração cortical e medular de desoxihemoglobina nos pacientes que desenvolveram NTA, que leva à diminuição do sinal de R2*, com perda da diferenciação entre essas regiões. Por outro lado, pacientes que desenvolvem RA apresentam queda da concentração de desoxihemoglobina na medula, o que leva a um aumento do sinal de R2*, permitindo boa diferenciação entre o córtex e a medula.

Tempos de eco inferiores aos relatados na literatura foram empregados neste estudo,^( 5 , 9 , 10 )^pois julgou-se que a sequência seria bastante sensível e permitiria a manutenção de resolução de contraste adequada independente da redução do TE, com a vantagem de diminuir o TR da sequência e, portanto, sua duração.

Em estudo que comparou pacientes com enxertos normais, NTA e RA, Han et al.,^( 2 )^mostraram que as diferenças de biodisponibilidade de oxigênio no rim transplantado nos estágios iniciais da disfunção de enxerto podem ser detectadas com eficiência, por meio dos valores R2* mensurados empregando-se o protocolo BOLD de RM.

Djamali et al.,^( 9 )^descreveram índices R2* inferiores em aloenxertos com NTA e RA comparados com aloenxertos com bom estado funcional, o que reflete um aumento significativo da biodisponibilidade de oxigênio na medula de aloenxertos com NTA. Entretanto, nesta análise, foram observados valores semelhantes de R2* entre os grupos NTA e Controle, embora mais baixos do que os do Grupo RA.

Dentre as limitações deste estudo, destaca-se o pequeno tamanho amostral. Outra limitação foi o fato de quatro pacientes terem necessitado de uma segunda biópsia em outro momento, ao longo das 4 semanas que sucederam o transplante, devido a alterações de seus achados clínicos. Esses pacientes foram submetidos a nova sequência BOLD de RM antes da segunda biópsia, o que pode ter introduzido um viés nesta análise. Além disso, uma minoria de pacientes (quatro casos) apresentou RA, e a variabilidade interobservador na estimativa do índice R2* não foi avaliada.

## CONCLUSÃO

Os resultados deste estudo indicam que o exame de ressonância magnética com sequência BOLD realizado em equipamentos de 3 Tesla é uma técnica viável e emprega mensurações indiretas da concentração tecidual de oxigênio para identificar lesões em enxertos renais. Futuramente, o uso rotineiro dessa sequência em casos de disfunção do enxerto renal pode vir a reduzir o número de biópsias nesses pacientes, que já se encontram debilitados, evitando possíveis complicações.
